# Clonidine for Management of Agitation in Delirious Patients

**DOI:** 10.1007/s11920-025-01617-5

**Published:** 2025-06-21

**Authors:** Dustin C. Rowland, Nicholas J. Waldvogel

**Affiliations:** Department of Psychiatry, University of Michigan, 4250 Plymouth Rd, Ann Arbor, MI 48105 USA

**Keywords:** Clonidine, Agitation, Antipsychotic tolerability, Treatment cost efficacy, Delirium

## Abstract

**Purpose of Review:**

This review explores clonidine as a potential treatment for agitation across various clinical contexts, focusing on its application in patients with limited cognitive reserve, hyperactive delirium, or conditions where standard treatments may exacerbate underlying symptoms, such as Parkinsonism. The review evaluates the pharmacological properties, efficacy, and practical considerations for using clonidine in managing agitation, including its role in other psychiatric and medical conditions.

**Recent Findings:**

Clonidine, a centrally acting alpha-2 adrenergic agonist, reduces norepinephrine release, leading to sedation, anxiolysis, and analgesia without significant respiratory depression. Recent studies and case reports highlight its utility in managing agitation, ADHD, autism spectrum disorders, dementia, and hyperactive delirium. Clonidine has demonstrated advantages over antipsychotics in preserving cognitive function and minimizing delirium duration. Additionally, it shows promise in treating PTSD-related nightmares and as an adjunct in anesthesia and analgesia.

**Summary/Takeaway:**

Clonidine offers a well-tolerated, cost-effective alternative to antipsychotics for managing agitation, particularly in vulnerable populations. Its diverse applications, favorable safety profile, and minimal impact on Parkinsonism make it a valuable tool in psychiatry and medicine. Further research is needed to refine dosing protocols and expand its indications in managing agitation and related symptoms.

## Introduction

Within the domain of consultation psychiatry, a common consult is for assistance with treatment of agitation. Agitation is a non-specific term that describes a wide range of behaviors. In patients with limited cognitive capacity secondary to acute or chronic traumatic insults– or other etiologies– agitation may represent non-cooperation with nursing staff. It may also be a sign of hyperactive delirium. Regardless of the underlying etiology, antipsychotics are commonly used for agitation; however, these have the untoward side effect of worsening Parkinsonism and may increase mortality in certain subsets of elderly patients. Many also have off-target anticholinergic effects that run the risk of precipitating or worsening delirium in patients with low cognitive reserve. There is a paucity of recent literature on other pharmacologic methods of agitation management, especially in patients with frontal encephalomalacia. We discuss the extant literature available on clonidine’s utilization in psychiatric and other domains in order to facilitate identification of scenarios in which this inexpensive and widely available drug may be helpful.

Management of agitation in patients with significant parkinsonism can be particularly difficult as medications favored for this indication (such as olanzapine and haloperidol) are highly anti-dopaminergic. One way to circumvent this problem is via antipsychotics that work primarily through serotonergic antagonism, such as quetiapine or pimavanserin. However, when these medications are ineffective, modulation of other neurotransmitters may be helpful.

Delirious patients have been noted to have elevated levels of norepinephrine, which contributes to one of the key features of delirium– inattention [1, 2]. Inattention is also a feature of ADHD, for which clonidine has been approved as a treatment (H). Modulation of norepinephrine release via clonidine has also been noted to be helpful for decreasing irritability and aggression in patients with autism spectrum disorders, Alzheimer’s disease, genetic intellectual disability, and bipolar mania [3–6].

Clonidine has sedative, anxiolytic, and analgesic properties due to its agonist activity at endogenous alpha receptors [7–9]. In addition, it is relatively inexpensive, well-tolerated, and has high oral bioavailability [7, 10]. Many of these properties are due to clonidine’s high affinity for alpha-2 over alpha-1 [11, 12]. It has a half life that varies widely between patients, with reported ranges from 5 to 25.5 h [1]. Metabolism is primarily through the hepatic CYP2D6 system, although some clonidine is excreted via the kidneys without undergoing metabolism [13]. Its activity at alpha 2 receptors results in decreased norepinephrine release and a resultant shift of autonomic activity towards the parasympathetic [14, 15]. The effects of this modulation are sedative, anxiolytic, and analgesic [7–9]. In addition, clonidine is relatively inexpensive, well-tolerated, and can be easily administered orally due to its high bioavailability [7, 10].

## Mechanism of Action

Clonidine is an alpha 2 agonist, similar to dexmedetomidine, that additionally binds to alpha 1 receptors with a much lower affinity [11]. Compared to dexmedetomidine, clonidine is less selective for alpha 2 receptors, with a 200:1 affinity ratio compared to the 1600:1 ratio of dexmedetomidine (M, U). However, it has been postulated that this difference has limited clinical relevance, with the differing effects of clonidine and dexmedetomidine resulting primarily from their pharmacokinetics [17]. Compared to the other commonly-used alpha 2 agonist, guanfacine, clonidine is “non-selective” against alpha 2 receptor subtypes, binding to A, B and C isoforms while guanfacine is selective for alpha 2A receptors [18]. Clonidine is metabolized primarily within the liver via CYP2D6, producing no active metabolites, but unmetabolized clonidine is also excreted by the kidneys [13]. 50% of clonidine is excreted in unmetabolized form by the kidneys while 50% is metabolized by the liver [18]. This results in a half life that varies widely based on individual patient characteristics, with a reported half life of between 5 and 25 hours. This is in contrast to dexmedetomidine, which has an average half life of 2 hours (U). When administered orally, clonidine reaches peak plasma concentration at 1 to 3 hours, depending on patient metabolism [1]. The oral bioavailability of clonidine is between 70% and 80%, compared to the < 20% oral bioavailability of dexmedetomidine, which requires buccal administration to reach similar bioavailability [7, 19]. In addition, clonidine is available in a 7-day patch formation in a variety of doses; which reduces fluctuations in plasma levels compared to oral or intermittent IV administration [20]. Interestingly, clonidine loses specificity for alpha 2 receptors at elevated blood concentrations and begins to bind to alpha 1 receptors; this allows it to preserve blood pressure when administered at sufficiently high doses.

Modulation of alpha receptors results in widespread effects at both central and peripheral locations. Within the central nervous system, stimulation of alpha 2 autoreceptors within the locus coeruleus reduces calcium channel activity, decreasing presynaptic release of norepinephrine (NE) (6,14,21*). Release of somatotroph hormone is elevated, increasing endogenous alpha 2 receptor activation (II). This has been postulated to reduce fear response to environmental stimuli, instead causing environmental indifference (ataraxia) [9, 17]. Reduction of overall NE release decreases autonomic tone, resulting in sedation and anxiolysis [1, 7–9, 15]. However, the respiratory center is not affected, allowing for sedation without risk of respiratory depression [21]*. Peripherally, clonidine increases NO release from endothelial cells, resulting in vasodilation and a reduction in capillary hydrostatic pressure– especially when applied topically [22, 23]. A global increase in parasympathetic tone and a reduction in inflammation is observed, resulting in a variety of effects, including cardiac sympathetic deactivation [1, 17]. Systemic levels of both renin and catecholamines are reduced [18]. Clonidine does produce analgesia, although the mechanism has not been well-delineated; part may be due to its peripheral effects on spinal cord glutamate release, complemented by the central effect of pain indifference (analgognosia) [17, 24]. The overall result of clonidine administration falls under the umbrella of “cooperative sedation”, which allows for sedation, anxiolysis, and analgesia without increasing the risk of delirium [17, 21*]. This wide range of effects has resulted in the use of clonidine for a large number of indications in addition to analgesia, including agitation, autism spectrum disorder, conduct disorder, mania, psychosis, delirium, nightmares and hypervigilance associated with PTSD, ADHD, opioid withdrawal, and others that are described below in Table 1.

**Table 1:** Clonidine dosing by indication

Indication	Dosing	Notes	Reference
ADHD, treatment ADHD, adjunctive treatment	PO: 0.1-0.4 mg/day. May divide into up to 4 separate doses Transdermal: 0.1-0.3 mg/day equivalent		[34, 42]
ADHD, adjunctive treatment, for children with comorbid ODD or conduct disorder	0.1-0.2 mg/day clonidine syrup		[3]
Agitation, associated with early onset-Alzheimer’s disease	0.1 mg BID	Case report	[4]
Akathisia	0.025 mg BID		[33]
Autism, hyperarousal associated with	0.1-0.3 mg/day		[34]
Behavioral disturbance, in autism spectrum or typically developing children	0.1-0.3 mg/day		[10]
TBI, severe, management of sympathetic hyperactivity	0.1 mg q12h		[43]
Tic disorder	Film: 1-2 mg/q7days; weight or age-based dosing	Equivalent to haloperidol in reducing tic scores; less side effects	[20, 44]
Delirium, hyperactive, critically ill patient	0.1-0.5 mg q6-8 h, PO		[11]
Delirium, older adult, after CABG	IV, loading: 0.5ug/kg IV, maintenance: 1-2ug/kg/hr	RCT where dexmedetomidine was found superior for ICU-critical end points	[16]
Delirium, prevention, after open heart surgery	Prior to surgery: 0.4ug/kg/hr After surgery: 0.2ug/kg/hr		[28]*
Delirium, subsyndromal	Day 1: 0.075 mg q3hrs, up to four doses Maintenance: 0.075 mg BID	To prevent progression to severe delirium	[31]
Dexmedetomidine, cross-titration to clonidine	0.1 mg; continue to increase every 6–8 h until patient is sufficiently sedated or blood pressure becomes limiting		[45]
Dexmedetomidine, withdrawal syndrome	0.1-0.3 mg q6-8 h		[12]
Dexmedetomidine withdrawal, taper	Days 1–4: 0.1 mg q6h Days 5–8: 0.05 mg q6h Days 9–12: 0.025 mg q6h		[7]
Mania, acute	0.1 mg BID	Case report; resource-poor area	[38]
Mania, acute, adjuvant to lithium	0.1-0.3 mg BID		[6]
Irritability associated with limited cognitive reserve	150ug-350ug		[46]
Korsakoff’s psychosis, improvement of attention	Day 1: 0.2 mg once Day 2: 0.2 mg BID Day 3: 0.2 mg TID Day 4: 0.2 mg QID		[47]
PTSD, childhood	Patch: 0.1-0.2 mg/24hrs		[41]
PTSD, childhood, aggression and nightmares	0.05 mg-0.1 mg TID		[40]
Sedation, critically ill adult	0.1-0.2 mg q6-8 h		[21]*
Sialorrhea, clozapine-induced	50ug/day	Case report; however, resulted in sexual disinhibition	[47]

**Human and Animal Rights**: This article does not contain any studies with human or animal subjects performed by any of the authors

### Anesthesia and Analgesia

The analgesic properties described above allow clonidine to be used as an adjunct to most pain regimens, reducing the use of deliriogenic medications [25]. When used in this manner for ICU analgesia, clonidine does not cause an increase in primary end points, such as vent time or overall length of stay, nor does it result in bradycardia or hypotension [9]. Clonidine has also been used as monotherapy for pain– most commonly in topical preparations where anti-inflammatory and vasodilatory effects can also aid in wound healing [22]. Its sympatholytic properties and effects on peripheral neurotransmission also make clonidine a reasonable topical treatment for neuropathic pain [27]. Some studies have noted that clonidine by itself has reasonable analgesic properties [26].

### Delirium

Although the general consensus on delirium is that actual treatment requires focus on underlying causes, it is not infrequent that hyperactive delirium results in a patient being at risk of self-injury or injury to others. Non-pharmacologic and environmental interventions are first-line, but acuity of presentation may necessitate pharmacologic intervention [2]. Sedating medications, such as antipsychotics, are often utilized to prevent harm, despite being associated with prolongation of delirium. Clonidine and other alpha-active agents are frequently used in delirium [28*]. Although any drug therapy can increase the duration of delirium, head to head comparison of clonidine with antipsychotics such as haloperidol demonstrate that clonidine results in a much milder average increase in delirium duration [29]*. Other trials have compared clonidine to other antipsychotics and sedatives, with the universal outcome of clonidine being less implicated in generation of persistent delirium [30]. Although the underlying etiology of delirium response is multifactorial, there is evidence that the agitation associated with delirium states may be linked to elevated levels of norepinephrine. Specifically targeting norepinephrine to reduce fear-learning response may reduce symptoms of hyperactivity while minimally prolonging delirium [1]. In addition, the effects of alpha 2 agonism on concentration may help focus and orient delirious patients by targeting inattention, a core feature of delirium [2]. Early clonidine intervention in subsyndromal delirium, targeting inattention and fear of environment, can prevent progression to a more dangerous hyperactive delirious state [31]. In addition, delirious patients with adverse reactions to antipsychotics, such as akathisia, can be treated with scheduled clonidine [33].

Only one well-designed study (The LUCID trial) was designed to evaluate clonidine’s effectiveness in treating delirium [32]. It was a double-blind, randomized, placebo-controlled trial which intended to enroll elderly patients, however, they were not able to recruit effectively due to exclusion criteria, and the study was halted. Further studies recruiting a wide pool of patients that meet criteria for inclusion are required.

### Agitation/Anxiety/Akathisia

Due to its nature as a sympatholytic, clinicians have used clonidine to target hyperactive symptoms such as anxiety and agitation in various settings. It has been used to improve akathisia, whether it is drug-related or not. For example, in one case of a woman with TBI and agitation complicated by risperidone-induced akathisia, clonidine 0.025 BID was successfully utilized to resolve akathisia, and was well-tolerated. Other studies have replicated this finding in this population [33]. It has also been shown to be well tolerated for agitation in other sensitive populations. For example, patient with TBI are sensitive to benzodiazepines and anticholinergics, so when considering an agent to treat akathisia, in a patient where beta-blockers may be contraindicated (such as those with asthma, diabetes, or COPD), reasonable options include bromocriptine or clonidine [18]. Another case describes a patient with Alzheimer Disease, a population notoriously sensitive to certain medications, who had a positive response to clonidine for agitation. Another study reported an Alzheimer patient that had a decreased requirement for benzodiazepines and restraints after administration of clonidine 0.1 mg BID, and a case of a patient with major neurocognitive disorder [4]. The transdermal formulation of clonidine is superior for anxiety, as compared to its oral form, the transdermal form provides a slow release of medication, thereby preventing the sedation from peak levels. It also avoids rebound anxiety related to rapid metabolism of the oral form. The transdermal formulation’s efficacy is distinct from the oral form [10].

### ADHD

Clonidine’s effectiveness in treatment of ADHD is well-established, touting Level 1 evidence. The long-acting formulation became FDA approved in 2010 after two 8-week trials. The first, a double-blinded, placebo-controlled study demonstrated significant improvement of ADHD symptoms in groups taking clonidine vs. placebo as scored on an ADHD symptom scoring tool. The second was an 8-week randomized, double-blind, placebo controlled, flexible dose study in children and adolescents. Participants were randomized to either take a stimulant alone or a stimulant with adjunctive clonidine. The adjunctive clonidine group showed significantly improved symptoms relative to the stimulant alone group as measured by an ADHD scoring tool [35]. Adding clonidine to stimulant treatment does not increase cardiovascular risk; it reduces incidence of insomnia in these patients (DDD). While effective for ADHD, a meta-analysis found that it had a moderate effect size of 0.58 +/- 0.16 on the symptoms of ADHD in children and adolescents, less than stimulants alone [36].

### Mania and Psychosis

Some studies have shown that clonidine has efficacy in treating mania and psychosis. Cognition and alertness in the CNS are theorized to be mediated by norepinepherine acting on postsynaptic alpha-2a receptors in the prefrontal cortex. However, chronic high doses of norepinepherine has been shown to decrease cognitive functioning, and it has been postulated that this mechanism may contribute to the cognitive decline as seen in schizophrenia. One translational study examined clonidine’s effect on electrophysiology parameters of those with schizophrenia. It reported that therapy increased mismatch negativity and P3a (of which low levels has been correlated to cognitive function in schizophrenia) in those with schizophrenia to mirror healthy controls. Further research in this area is underway. Clinically, there are reports of clonidine use benefitting those with mania. For example, one case of a young man suffering from mania who was not responding to four weeks of inpatient antipsychotic therapy, and had required restraints, had an excellent response to clonidine, resolving his mania in two days [37]. It has evidence that it may be beneficial in patients with mania as an adjunct to lithium as well, and has been used in management of mania since the 1980’s. One randomized, double-blind, placebo-controlled study of 90 patients undergoing treatment with mania were randomized to lithium monotherapy plus placebo or a lithium plus clonidine group. Results showed improved sleep and symptom severity in those treated with lithium plus clonidine relative to lithium plus placebo [6].

### PTSD Nightmares

Only case reports have studied clonidine’s effectiveness in treating PTSD nightmares, and results are generally positive. There are a two postulated theories as to why clonidine is useful. One theory is that it decreases traumatic memory consolidation by decreasing basolateral amygdala hyperactivation [38]. Another relies on its effect on sleep architecture: it has been shown to alter REM and NREM sleep. At low doses (0.025 mg), it increases REM and decreases NREM sleep, while at high doses (> 0.15 mg) it increases NREM and decreases REM sleep. Multiple case reports have found clonidine to be effective in treating nightmares in PTSD, and postulate it should be examined more as an adjunct to prazosin [8, 39]. One case study reported that in an 18 year old boy with severe PTSD and insomnia, transdermal clonidine was initiated and uptitrated to 0.2 mg/24 h as an adjunct to other pharmacotherapy. Within one month of this addition, his insomnia resolved, and his Beck Depression Inventory Score decreased from 28 to 7 without any other substantial intervention [40].

### Anesthesia/Analgesia

Clonidine has well-established efficacy in its use as an anesthetic. It has been shown to decrease the dose requirement of other analgesics, including benzodiazepines, to be utilized. It also has been shown to decrease emergence agitation. In a randomized trial studying post-anesthesia emergence agitation in children treated with sevoflurane vs. sevoflurane and clonidine, the absolute risk reduction of emergence agitation in those treated with sevolflurane and clonidine decreased by 52.9% [25]. Topical clonidine may have use for neuropathic pain. When used topically, clonidine promotes vasodilation by decreasing norepinephrine release and thereby improving microvascular dysfunction. A double-blind, placebo-controlled trial studying topical clonidine/pentoxyifylline to treat neuropathic pain is currently underway [22].

## Conclusion

Clonidine is a versatile, well-tolerated, and cost-effective medication with broad utility in managing agitation and associated conditions across diverse patient populations. Its specific pharmacological properties, including alpha-2 adrenergic agonism, enable it to provide sedation, anxiolysis, and analgesia while preserving respiratory function and minimizing the risks associated with antipsychotics, such as exacerbation of Parkinsonism or prolongation of delirium. Clonidine’s inexpensive oral formulation makes it an accessible option in resource-limited settings, and its use is associated with a lower likelihood of prolonged hospital stays compared to antipsychotics. Beyond agitation, clonidine demonstrates efficacy in treating conditions such as ADHD, autism spectrum disorders, PTSD-related nightmares, and hyperactive delirium, which allows it to be used in situations where diagnostic clarity has not yet been attained. Despite its established role in certain domains, there remains a need for more robust research to clarify optimal dosing strategies and expand its evidence base, particularly in populations with cognitive impairments or complex medical conditions. Clonidine represents an invaluable tool for clinicians seeking effective and safer alternatives for managing agitation and related symptoms.

## Data Availability

No datasets were generated or analysed during the current study.

## Key References

- Purivatra E, Guenette M, Coleman B, Cheung A, Burry L. High-versus low-dose clonidine for sedation and analgesia in critically ill adults: A retrospective cohort study. *J Clin Pharm Ther.* 2021;46(6):1706–13. **Evaluates dosing strategies for clonidine in critically ill adults**,** offering practical insights into its use for sedation and analgesia.**
- Smit, L., Dijkstra-Kersten, S. M. A., Zaal, I. J., van der Jagt, M., & Slooter, A. J. C. (2021). Haloperidol, clonidine and resolution of delirium in critically ill patients: a prospective cohort study. *Intensive Care Medicine*, 47, 316–324. **This study compares clonidine and haloperidol**,** providing direct evidence of clonidine’s advantages in resolving delirium with fewer adverse effects**,** making it central to agitation management discussions**.
- Neerland BE, Busund R, Haaverstad R, Helbostad JL, Landsverk SA, Martinaityte I, et al. Alpha-2-adrenergic receptor agonists for the prevention of delirium and cognitive decline after open heart surgery (ALPHA2PREVENT): protocol for a multicentre randomised controlled trial. *BMJ Open.* 2022;12(6):e057460. **Protocol for a multicenter trial exploring alpha-2 agonists for preventing delirium and cognitive decline after surgery**,** highlighting clonidine’s preventive potential. Establishes a foundation for future large-scale studies on clonidine’s efficacy**.
